# The Comparison of the Profile of Phenolic Compounds in Noni (*Morinda citrifolia* L.) Fruit by Different Drying Methods

**DOI:** 10.3390/foods14081398

**Published:** 2025-04-17

**Authors:** Qianxin Li, Juan Chai, Shenghui Deng, Jucai Xu, Yanxian Feng, Ruili Yang, Wu Li

**Affiliations:** 1 Guangdong Provincial Key Laboratory of Large Animal Models for Biomedicine, School of Pharmacy and Food Engineering, Wuyi University, Jiangmen 529020, China; 13426768070@163.com (Q.L.); 18185331627@163.com (S.D.); xujucai.happy@163.com (J.X.); yanxianfeng@wyu.edu.cn (Y.F.); 2 Key Laboratory of Food Nutrition and Functional Food of Hainan Province, College of Food Science and Engineering, Hainan University, Haikou 570228, China; 15152362806@163.com; 3 College of Food Science, South China Agricultural University, Guangzhou 510642, China; rlyang77@scau.edu.cn

**Keywords:** noni, drying methods, polyphenols, antioxidant activity

## Abstract

In this study, the compositional shifts in free and bound phenolic compounds of *Morinda citrifolia* L. (Noni) processed by different drying methods were investigated. Twenty-seven phenolic compounds, predominantly rutin and quinic acid, were discovered in fresh Noni fruit. Vacuum freeze-drying retained the highest free phenolic content, with rutin (1809.83 mg/kg DW) and quinic acid (198.72 mg/kg DW) as the primary constituents, while bound phenolics were dominated by benzoic acid (35.56 mg/kg DW). Hot-air drying reduced the free phenolics by 51.59% (80% methanol) and the bound phenolics (base hydrolysis) by 35.55%, with a significant degradation of rutin and quinic acid. Microwave drying similarly decreased the free phenolics, though the caffeic acid increased to 46.45 mg/kg DW due to the thermal stability. Bound phenolics showed the highest content (alkaline hydrolysis) in fresh fruits, primarily benzoic acid (220.67 mg/kg DW) and rutin (77.02 mg/kg DW), surpassing the acid/enzyme methods. While vacuum freeze-drying effectively preserved the free phenolics, thermal methods (hot-air/microwave drying) promoted the release of quercetin (free phenols) and 3,4-dihydroxybenzoic acid (bound phenols). The findings of this study elucidate the species-specific compositional dynamics of phenolic compounds under different drying regimes, while providing quantitative guidelines for advancing the understanding of the underlying health-promoting phytochemical profiles of Noni.

## 1. Introduction

Polyphenols, a diverse group of phytochemicals abundant in plant-based foods, are renowned for their antioxidant, anti-inflammatory, and health-promoting properties. Based on solubility and structural associations within the matrix, polyphenols are classified into free and bound forms. Free phenolics are primarily localized within cellular vesicles and are readily extractable, while bound phenolics are esterified or glycosylated to cell wall components, requiring acid or enzymatic hydrolysis for release [1]. While both forms exhibit significant antioxidant capacity and health-promoting properties, their physiological effects vary markedly depending on variations in the molecular composition, bioavailability, metabolic pathways, and biotransformation by gut microbiota [2,3]. Free phenolic compounds, characterized by a low molecular weight and polarity, are generally rapidly absorbed in the small intestine, exerting immediate antioxidant effects [4]. In contrast, bound phenolics require hydrolysis by colonic microbiota to release bioactive aglycones, resulting in delayed but sustained antioxidant and anti-inflammatory activities [5]. Additionally, variations in the composition of polyphenol monomers critically determine their physiological effects by modulating interactions with specific molecular targets [6,7].

Due to their molecular structure, polyphenols are highly susceptible to processing conditions. The composition of polyphenols often undergoes substantial modifications during processing, leading to corresponding shifts in the health benefits associated with polyphenol consumption. Previous research has demonstrated that the thermal processing of fruits and vegetables can modify the levels of polyphenols, which can enhance or reduce their cardiovascular benefits, depending on the type of processing and the compositional changes [8]. The processing can induce microstructural changes in the food matrix, potentially increasing the extractability and bioavailability of polyphenols [9], and influencing the interaction between dietary polyphenols and the gut microbiome, ultimately affecting their health-promoting effects [10]. These findings underscore the necessity of evaluating processing-induced compositional changes when assessing the functionality of food polyphenols.

Noni (*Morinda citrifolia* L.), a tropical plant traditionally revered in Polynesian and Southeast Asian medicine, is increasingly recognized as a “superfruit” due to its rich bioactive compounds, particularly polyphenols [11]. Recent phytochemical analyses have identified over 20 phenolic compounds in Noni fruits, including flavonoids, phenolic acids, and lignans [11,12]. Comparative studies indicate that the relative concentrations of specific flavonoids (e.g., quercetin, kaempferol, and rutin) and phenolic acids (e.g., chlorogenic acid, coumaric acid, and gallic acid) in Noni fruits differ significantly from those in common tropical fruits, such as mangoes, avocados, and apples. This distinct composition likely underlies Noni fruit’s unique bioactive profile, particularly its enhanced antioxidant, antibacterial, and anti-inflammatory activities [13]. Notably, the stability and bioavailability of these phytochemicals are substantially affected by postharvest processing techniques, with drying methods playing a critical role in determining the phenolic preservation and extraction efficiency. Freeze-drying has demonstrated the superior retention of heat-sensitive polyphenols compared to conventional hot-air drying in several fruits [14,15]. Contrastingly, studies on lemon (*Citrus limon*) pomace revealed hot-air drying as yielding the highest total polyphenols and antioxidant activity compared to freeze-drying [16]. Similarly, microwave drying yielded the highest polyphenol content and antioxidant activity compared with oven-drying and freeze-drying in the drying of *Capparis spinosa* L. fruits [17]. These divergences underscore the species-specific responses. Previous studies on Noni and Noni powder have shown that drying leads to a decrease in the total phenolic compounds and antioxidant activity of Noni [18,19]. Despite these advances, systematic comparisons of changes in the polyphenol composition, especially in bound and free phenols, under different drying regimes remain limited. We hypothesize that different drying methods may affect the compositional profiles of free versus bound phenolic compounds in Noni fruit, leading to significant variations in their bioactivity. Given the processing-dependent fluctuations in the polyphenol composition, analyzing the post-drying changes in the free and bound phenolic profiles is essential for optimizing Noni-based product efficacy and validating their health benefits.

Thus, in this study, we compared the changes in the composition and antioxidant activity of both free and bound phenols, using different extraction methods, in Noni dried by hot-air, microwave, and vacuum freeze-drying methods. The findings of this study provide critical insights for the development and application of Noni and Noni-based products, while advancing the scientific understanding of the underlying Noni health-promoting phytochemical profiles.

## 2. Materials and Methods

### 2.1. Chemicals and Reagents

The standards, including quinic acid, phloroglucinol, gallic acid, helicid, 3,4-dihydroxybenzoic acid, p-hydroxybenzoic acid, esculetin, caffeic acid, vanillic acid, syringic acid, benzoic acid, rutin, p-coumaric acid, vanillin, p-hydroxycinnamic acid, hyperoside, isoquercitrin, ferulic acid, kaempferol-3-o-rutinoside, isoferulic acid, kaempferol-3-o-glucoside, hesperidin, salicylic acid, morin, quercetin, cinnamic acid, kaempferol, acetonitrile and formic acid (HPLC grade), 1,1-diphenyl-2-picrylhydrazyl (DPPH), 2,4,6-tri (pyridin-2-yl)-1,3,5-triazine (TPTZ), 2-azino-bis (3-ethylbenzothiazoline-6-sulfonic acid) diammonium salt (ABTS), and 6-hydroxy-2,5,7,8-tetramethylchroman-2-carboxylic acid (Trolox), were procured from Adamas Reagent, Ltd. (Shanghai, China). Cellulase (400 µ/mg), hemicellulose (20,000 µ/g), and pectinase (500 µ/mg) were acquired from Shanghai Yuanye Bio-Technology Co., Ltd. (Shanghai, China).

### 2.2. Noni Materials

Noni fruits were collected from the Noni Planting Base in Wanning in Hainan Province, China. Mature Noni fruits that were morphologically perfect were taken for the following treatment: the Noni fruits were rinsed and sliced (to a thickness of 1.5 cm), and then separated in three parts. Hot-air drying was conducted using a GZX-9076MBE oven (Boxun Instruments, Shanghai, China) at 60 °C. Microwave drying employed an M1-L213B system (Midea Group, Foshan, Guangdong, China) with a 600 W power and 150 g sample loading. Freeze-drying used an LGJ-12A lyophilizer (Beijing Sihuan Qihang Co., Ltd., Beijing, China) at −50 °C under a vacuum (≤20 Pa). All of the drying processes were terminated when the sample moisture content reached 10% (w.b.). The dried samples were deseeded, ground using a grinder, and sieved through a 100 μm mesh screen to obtain the hot-air drying, microwave drying, and vacuum freeze-drying samples. The seeds from the fresh fruits were removed and homogenized as fresh fruit samples. Then, all of the samples were stored at −20 °C for subsequent uses.

### 2.3. Extraction of Polyphenols

#### 2.3.1. Extraction of Free Polyphenols

Free polyphenols were extracted mainly by water or 80% methanol (1% formic acid) solvent. The extraction method followed the previously described studies [20]. Briefly, polyphenols were extracted from 1.00 g of Noni with 30 mL of 80% methanol (including 1% formic acid) (*v*/*v*) at 320 W and 25 °C for 30 min in an ultrasonic cleaner. The mixture was centrifuged for 15 min at 11,000 r/min. This procedure was repeated three times. Then, the supernatants were pooled and concentrated by a rotary evaporator at 45 °C to avoid light and resuspended with 70% methanol to 10 mL. The method of using water as an extraction solvent was performed as described above.

#### 2.3.2. Extraction of Bound Polyphenols

Bound polyphenols were extracted by acid hydrolysis, base hydrolysis, and enzyme hydrolysis. The method of acid hydrolysis was employed according to a previous study with some changes [20]. Briefly, the residue (1.00 g) after extracting the free polyphenols by 80% methanol was conditioned with 30 mL of HCl (3 mol/L) at 85 °C for 60 min in a water bath; then, the reaction mixture was acidified to a pH = 2 with NaOH (10 mol/L) and centrifuged at 11,000 r/min for 20 min. The supernatant was extracted three times with 35 mL of ethyl acetate. Then, the ethyl acetate layers were combined and concentrated by a rotary evaporator at 45 °C until dryness, and, finally, 70% methanol was added up to 10 mL. Base hydrolysis was obtained according to Tang et al. [21]. Bound polyphenols (1.00 g of residue after the free polyphenol extraction) were extracted with a 30 mL NaOH (10 mol/L) solution including EDTA-2Na (10 m mol/L) and 1% ascorbic acid in a shaking bath at 30 °C for 4 h, and brought to a pH = 2 with HCl (6 mol/L). The mixture was centrifuged at 11,000 r/min for 20 min. The supernatant was extracted three times with 35 mL of ethyl acetate, concentrated, and then dissolved, similar to the procedure for acid hydrolysis. The enzymes consisted of hemicellulose, cellulase, and pectinase. An amount of 30 mL of H_2_O, which was adjusted to a pH of 5 by citric acid and 0.08 g of compound enzymes (hemicellulase/cellulase/pectinase = 2:1:1), was added to the Noni residue (1.00 g), heated at 50 °C for 2 h in a shaking bath, and subsequently sonicated for 30 min in an ultrasonic cleaner. The extraction mixture was rapidly cooled to room temperature and centrifuged at 11,000 r/min for 20 min; then, the supernatant was extracted and concentrated, and, finally, the resulting residue was solubilized to 10 mL with 70% methanol.

### 2.4. Total Polyphenol Content (TPC)

The TPC was estimated by the Folin–Ciocalteu colorimetric method, as reported previously [22]. In this work, 125 μL of the extracts after proper dilutions were added to 500 μL of H_2_O and 125 μL of Folin–Ciocalteu reagent, fully blended, and left to stand for 6 min at room temperature; then, 1.25 mL of 7% Na_2_CO_3_ solution and 1 mL of H_2_O were transferred into the reaction tube. The mixture was incubated at 30 °C for 90 min in the dark. Upon completion, the absorbance of the samples was measured at 760 nm. The concentration of polyphenols was assessed by gallic acid as the standard, and the concentration range was 10~100 µg/mL (R^2^ = 0.998). The results were expressed with mg gallic acid equivalents/g dry weight (mg GAE/g DW) of the Noni sample.

### 2.5. UPLC-Q-TOF-MS Analysis

The Agilent ZORBAX Eclipse Plus C18 (2.1 × 100 mm, 1.8 μm) column was used for the separation of the polyphenols. In this study, 0.1% formic acid in water and acetonitrile consisting of mobile phases A and B were used. The elution program was formed as follows: 0~3 min at 5~15% B, 3~11 min at 15%~30% B, 11~15 min at 30%~50% B, 15~21 min from 50% to 90% B, and 21~22 min from 90% to 5% B. The flow rate was set at 0.15 mL/min, the column temperature was set at 40 °C, and the sample size was 2 µL. The Q-Exactive Orbitrap MS (Thermofisher Scientific, Beijing, China) coupled to an electrospray ionization (ESI) source was applied to the eluent compounds. The MS spectra were set in the positive and negative modes, with a range between 100 and 1500 *m*/*z*. The capillary voltage was 3200 V. Nitrogen was used with a sheath gas flow of 35 arb and an aux gas flow of 10 arb.

By comparing the spectra and retention times of the sample with the external standards, the following polyphenols were identified: quinic acid, phloroglucinol, gallic acid, helicid, 3,4-dihydroxybenzoic acid, p-hydroxybenzoic acid, esculetin, caffeic acid, vanillic acid, syringic acid, benzoic acid, rutin, p-coumaric acid, vanillin, p-hydroxycinnamic acid, hyperoside, isoquercitrin, ferulic acid, kaempferol-3-o-rutinoside, isoferulic acid, kaempferol-3-o-glucoside, hesperidin, salicylic acid, morin, quercetin, cinnamic acid, and kaempferol. The external standard curves were made to quantify the polyphenols. The range of the correlation coefficients (r^2^) was 0.9984–0.9999, the linear ranges were (ng/mL) 11.424~5025.794, the LOD (ng/mL) was 5.362~49.897, the LOQ (ng/mL) was 17.873~166.323, and the intra-day precision was RSD < 3% and the inter-day precision was RSD < 5%.

### 2.6. Antioxidant Activity

The ABTS assay was determined according to the method of Van der Werf et al. [23]. In short, 176 μL of 140 mmol/L potassium persulfate solution was added to 10 mL of 7 mmol/L ABTS solution and incubated for 12~16 h at room temperature while being protected from light. Subsequently, the stock solution was adjusted to the absorbance, which was 0.7 ± 0.02 at 734 nm, by H_2_O; then, 200 μL of the sample after appropriate dilutions was mixed with 4 mL of diluted ABTS^+^ stock solution and reacted for 6 min in darkness at room temperature; the absorbance was read at 734 nm. For this investigation, the standard curve was computed with Trolox, and the concentration was between 10 and 100 μg/mL (R^2^ = 0.999). The results were quantified as µmol Trolox equivalents/g dry weight (µmol TE/g DW) of the sample.

The FRAP assay was conducted using the method by Sompong et al. [24], with some changes. The FRAP solution was manufactured freshly and consisted of acetate buffer (300 mM; pH = 3.6), TPTZ (10 mmol/L), and FeCl_3_ (20 mmol/L) at 10:1:1 (*v*/*v*/*v*), and was pre-incubated at 37 °C in a water bath. Thereafter, 30 μL of the sample after suitable dilutions was added to 900 μL of FRAP solution and reacted for 30 min at room temperature while being protected from light; the absorbance was read at 593 nm. The standard curve was computed with FeSO_4_·7H_2_O and the concentration ranged from 0.1 to 2.0 mmol/L (R^2^ = 0.999). The results were expressed as µmol ferrous sulfate equivalents/g dry weight (µmol Fe (II) SE/g DW) of the sample.

The DPPH assay was measured based on the Goyal et al. [25] method. Briefly, 50 µL of the sample after appropriate dilutions was reacted with 400 µL of DPPH (100 µmol/L) at room temperature for 30 min, and the absorbance was read at 517 nm. The results were expressed in µmol TE/g DW and were calculated using Trolox (10~150 µg/mL) as the standard curve (R^2^ = 0.997).

### 2.7. Data Analysis

Data were collected three times and presented as mean ± standard deviation, and were evaluated with the ANOVA test (Tukey’s and Bonferroni) for significant differences at a *p* < 0.05 level. SPSS (version 20.0) was used for the statistical analyses.

## 3. Results and Discussion

### 3.1. The TPC of Noni

**Figure 1:**
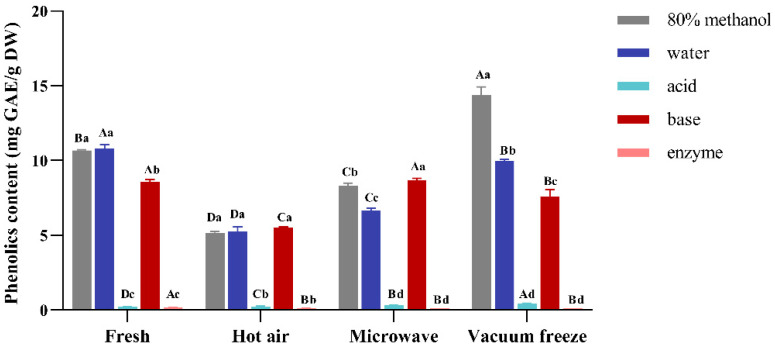
Total polyphenol content of Noni with different drying methods. A–D: statistically significant differences between the different drying methods; a–d: statistically significant differences between the different extraction methods.

The TPC of Noni using different drying methods is summarized in Figure 1. For fresh fruits, the free polyphenols extracted by 80% methanol and water were 10.68 mg GAE/g DW and 10.81 mg GAE/g DW, while the bound polyphenols extracted by acid, base, and enzyme were 0.21 mg GAE/g DW, 8.55 mg GAE/g DW, and 0.18 mg GAE/g DW, respectively. Compared with the fresh fruits, hot-air and microwave drying showed a significant reduction, and the content of polyphenols extracted by hot-air drying was significantly lower than that by microwave. In hot-air drying, the free polyphenols extracted by 80% methanol and water (51.59% and 51.24%, respectively), and the bound polyphenols extracted by base decreased by 35.55%. In microwave drying, the bound polyphenols extracted by base showed no difference. Vacuum freeze-drying had the highest amounts of free polyphenols through the 80% methanol extraction (14.38 mg GAE/g DW).

Our results confirmed that hot-air drying had the greatest impact on the TPC. Hot-air drying methods often induce significant polyphenol degradation through oxidative reactions, enzymatic browning, and Maillard reactions [26,27]. In addition, the rate of oxidation and degradation of polyphenolic compounds becomes faster with increasing temperature and time [28]. Conversely, low-temperature drying methods like freeze-drying preserve the hydroxyl group integrity by sublimating water under vacuum, thereby mitigating thermal decomposition [29,30]. Emerging evidence also suggests that vacuum drying, which produces rapid moisture removal with reduced oxygen exposure, may limit the oxidation of redox-sensitive phenolics [9]. The free polyphenols decreased after microwave drying, which might be due to the thermal degradation of polyphenolic compounds induced under high-power conditions, as well as being related to Meladic browning. In addition, microwave drying may cause physical damage to the cellular structure of the fruit, resulting in the loss of intracellular polyphenol release [31]. Moreover, vacuum freeze-drying displayed significantly higher polyphenols compared to the fresh fruit by 80% methanol extraction. The reason for such a difference might be associated with the structural changes and inactive enzymatic activity caused by the impeded browning reaction [32,33]. Vacuum freeze-drying enhances the extractability of phenolic compounds because it effectively releases low-molecular-weight phenolics [34] while simultaneously suppressing the activity of polyphenol oxidase under low-temperature vacuum conditions, thereby effectively reducing the oxidative degradation of polyphenols [14]. In addition, our results indicate that bound polyphenols may be freely released under vacuum freeze-drying. Furthermore, the different extraction methods significantly affected the content of bound polyphenols; the content of polyphenols via acid and enzyme hydrolysis were lower than with base hydrolysis, which was comparable with the findings of Tang et al. [21]. Compared to alkali hydrolysis, acid hydrolysis requires higher temperatures, which, combined with highly acidic conditions and prolonged hydrolysis, results in the loss of polyphenol degradation [35,36]. In addition, under acidic conditions, the hydroxyl groups in polyphenols are easily protonated, which breaks the polyphenol molecule and releases monomers or oligosaccharides. In addition, some non-polyphenol-reducing substances, such as organic acids and amino acids, may also be present in polyphenol extracts and react with the Folin–Ciocalteu reagent, resulting in differences [37,38,39].

### 3.2. Identification of Polyphenols in Noni Fruit

A total of 27 polyphenols were characterized through the mass spectrometry data and references (Table 1). Among them, 21 polyphenols were directly identified according to the retention time, *m*/*z*, and secondary *m*/*z* fragments of the standard, including phloroglucinol, gallic acid, helicid, 3,4-dihydroxybenzoic acid, p-hydroxybenzoic acid, caffeic acid, vanillic acid, syringic acid, benzoic acid, rutin, p-coumaric acid, vanillin, p-hydroxycinnamic acid, hyperoside, isoquercitrin, ferulic acid, isoferulic acid, kaempferol-3-o-glucoside, quercetin, cinnamic acid, and kaempferol. Compound 1 was identified as quinic acid by the parent ion at *m*/*z* 191.0561 (M-H) and the created MS/MS fragment ion at *m*/*z* 127.0401 [(M-H)-2CH_2_OH]^−^. Compound 7 had a parent ion at *m*/*z* 177.0183 (M-H) and generated a fragment ion at *m*/*z* 133.0284 [(M-H)-CO_2_]^−^, thus corresponding to esculetin. Compound 27 gave *m*/*z* 285.0404 (M-H), and compound 19 was similar to kaempferoside. The parent ion was *m*/*z* 593.1514 (M-H), and 308 mass units were lost from the parent compound, which may be the loss of a hexose and a deoxyhexose, tentatively identified as kaempferol-3-o-rutinoside [39]. Compound 22 was confirmed as hesperidin by exhibiting *m*/*z* 609.1829 (M-H) and fragment ion at *m*/*z* 301.0723. Compound 23 was identified as salicylic acid, which was confirmed by the parent ion with *m*/*z* 137.0230 (M-H). Compound 24 was easily characterized as morin with parent ion at *m*/*z* 301.0359, and fragment ions at *m*/*z* 151.0028. The chromatograms are shown in the Supplementary Material (Figures S1–S4).

### 3.3. Quantity of the Polyphenols in Noni Fruit by Different Drying Methods

The contents of polyphenols in fresh fruits are quantified in Table 2. The results revealed that the total free polyphenol contents presented higher values compared to the bound polyphenols. For the free polyphenols, rutin showed the highest content, followed by quinic acid, quercetin, and kaempferol-3-o-rutinoside. Base hydrolysis (484.15 mg/kg DW) released the highest polyphenol contents when compared to acid and enzyme hydrolysis, and the polyphenol contents of the fresh fruits followed the decreasing order of benzoic acid > rutin > isoferulic acid. Benzoic acid, p-coumaric acid, p-hydroxycinnamic acid, and vanillin were not detected in free polyphenols of the fresh fruits, while morin, hesperidin, helicid, and esculetin were not detected in the bound polyphenols.

Dussossoy et al. [40] reported that desacetylasperulosidic acid showed the highest number of polyphenols in Costa Rican Noni juice, followed by asperulosidic acid and rutin; Gironés-Vilaplana et al. [41] verified that the predominant polyphenols in Noni fruit were lucidin, quercetin-3-o-rutinoside, and kaempferol-3-o-rutinoside. There is little literature on the identification of polyphenols in Noni fruit, and the treatments and polyphenol extraction methods varied widely, so these reports were inconsistent with our results. This also may be related to the cultivars, cultivated regions, and maturation stage.

The polyphenol contents by hot-air drying in Noni are shown in Table 3. Specifically, the bound polyphenols and free polyphenol contents by 80% methanol hydrolysis were dominant compared to the fresh fruits. Rutin, quinic acid, kaempferol-3-o-rutinoside, and benzoic acid were the predominant substances in the free polyphenols, and benzoic acid, isoferulic acid, and rutin played a role in the bound polyphenols. Gallic acid, p-coumaric acid, kaempferol, and p-hydroxycinnamic acid were only detected in the bound polyphenols, and caffeic acid, quercetin, helicid, esculetin, and hesperidin were only detected in the free polyphenols. Our results pointed out that the corresponding free and bound polyphenols significantly declined during hot-air drying compared to the fresh fruits. The free polyphenols, including rutin, quercetin, 3,4-dihydroxybenzoic acid, and kaempferol-3-o-rutinoside, were decreased significantly during the 80% methanol extraction, and benzoic acid, rutin, p-coumaric acid, and isoferulic acid were lower than the fresh fruits in bound polyphenols. This change was consistent with the TPC (see Section 3.1). This discrepancy can be explained by the high temperature and long drying time, which led to the destruction of the cell wall. The released oxidase and hydrolase can degrade the polyphenols [31].

As shown in Table 4, the free polyphenols were the most abundant in microwave drying. Regardless of the extraction methods, the most abundant was rutin in the free polyphenols, followed by quinic acid and caffeic acid. Benzoic acid was detected in high concentrations in the bound polyphenols, and then isoferulic acid, 3,4-dihydroxybenzoic acid, and other polyphenols, such as quinic acid, were the main polyphenols. In our study, the contents of polyphenols detected by microwave drying were significantly lower than that of fresh fruits. This can be explained by the loss of thermolabile antioxidants caused by the temperature and oxidation [42,43]. Among them, p-coumaric acid, cinnamic acid, and p-hydroxycinnamic acid were only detected in the bound polyphenols, while caffeic acid was only detected in the free polyphenols, and the contents increased significantly. This differential distribution may be attributed to caffeic acid’s exceptional thermal stability and resistance to polyphenol oxidase-mediated catabolism [17,42,43]. Saha et al. [44] discovered that microwave drying possessed the highest level of caffeic acid in corncob compared to hot-air drying and freeze-drying, but the reasons need to be further studied. During the microwave drying, rutin was the most degraded, followed by quinic acid, quercetin, and 3,4-dihydroxybenzoic acid. This is in accordance with the results of Saha et al. [44], and rutin may be degraded into simple polyphenols, which attenuated the thermal degradation so that other polyphenols had a lower variation during drying.

The effect of vacuum freeze-drying on the polyphenols is shown in Table 5. Observing the polyphenol contents, the free polyphenols by 80% methanol hydrolysis showed higher amounts than in the fresh fruits. Rutin and quinic acid were the highest amounts in the free polyphenols, followed by kaempferol-3-o-rutinoside, hyperoside, and isoquercitrin. The bound polyphenols had great significance through the different hydrolysis methods. In acid hydrolysis, the polyphenol contents were presented as benzoic acid > 3,4-dihydroxybenzoic acid > quinic acid. Benzoic acid, isoferulic acid, rutin, and p-coumaric acid were the main polyphenols using base hydrolysis. Benzoic acid, quinic acid, rutin, and isoferulic acid were dominant with enzyme hydrolysis. Kaempferol-3-o-glucoside, helicid, and morin were only detected in the free polyphenols, and caffeic acid, p-coumaric acid, cinnamic acid, and p-hydroxycinnamic acid were not detected. This study indicated that the polyphenols by vacuum freeze-drying were significantly higher than hot-air and microwave drying, especially the free polyphenols extracted by 80% methanol. The results showed that vacuum freeze-drying can better preserve the polyphenols in Noni. The vacuum freeze-drying usually prevented the degradation of heat or oxygen-sensitive bioactive compounds [31]. Thus, the higher phenolic contents extracted with 80% methanol were likely due to structural matrix modifications and enzymatic inactivation induced by vacuum freeze-drying [32,45].

### 3.4. The Changes in Antioxidant Activity

**Figure 2:**
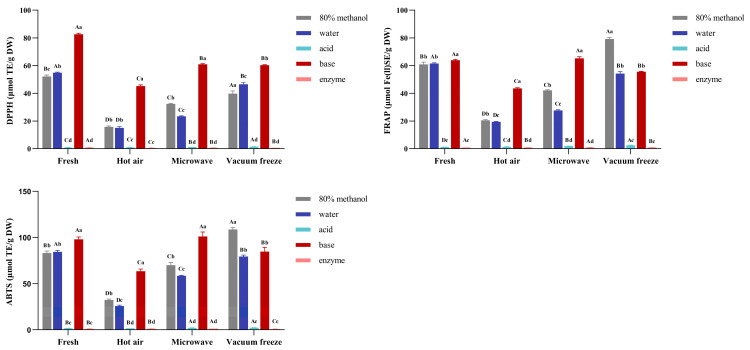
The antioxidant activity of Noni fruit. A–D: statistically significant differences between the different drying methods; a–d: statistically significant differences between the different extraction methods.

In this research, we focused on the changes in the antioxidant activity in Noni using different drying methods. As shown in Figure 2, for different extraction methods, 80% methanol extractions possessed the highest antioxidant activity, with 79.27 μmol FeSO_4_/g DM and 108.72 μmol TE/g DW by the FRAP and ABTS methods with vacuum freeze-drying, followed by base hydrolysis and water extraction. These results were consistent with the contents of the polyphenols, and the high contents of rutin played an important role in the antioxidant activity [45]. The highest antioxidant activity was observed in base-hydrolyzed bound phenolics, despite vacuum freeze-drying showing superior free phenolic retention, which may stem from the rapid interaction between specific base-released antioxidants (e.g., benzoic acid derivatives) and peroxyl radicals, which contrasted with the steric hindrance-induced sluggish reaction of glycosylated compounds, like rutin in the DPPH assays [46,47]. This disparity is further complicated by the potential interference from non-phenolic components (e.g., aromatic amino acids and glutathione) in DPPH systems [47]. Except for the vacuum freeze-drying, bound polyphenols using base hydrolysis exhibited stronger antioxidant activity compared with the free polyphenols, which could be a result of the isoferulic acid and benzoic acid showing stronger antioxidant activity because of their structural characteristics and the type and quantity of substituents [48,49,50]. Meanwhile, the polyphenols were not the only influencing factor in the antioxidant activity, and other phytochemicals may also contribute, such as organic acids [51]. This study demonstrated that the antioxidant activity of polyphenols was influenced by different drying methods in Noni. Fresh fruit and vacuum freeze-drying involved good antioxidant activity, and vacuum freeze-drying using 80% methanol extraction had a better antioxidant capacity. Hot-air and microwave drying led to a significant decrease in the antioxidant activity of Noni, which may be related to the reduced rutin; the thermal degradation or consumption by antioxidants in the Maillard reaction pathway may also have caused the lower antioxidant activity [44,52].

Our findings demonstrated the distinct impacts of vacuum freeze-drying, hot-air drying, and microwave drying on the phenolic profiles of Noni fruit. The highest levels of free phenolics, notably, rutin (1809.83 mg/kg DW) and quinic acid (198.72 mg/kg DW), were observed after vacuum freeze-drying, which was consistent with a previous study showing that low-temperature drying minimized the thermal degradation and enzymatic oxidation of phenolics in papaya fruit and cocoa [15,31]. In contrast, microwave drying resulted in a 51.59% reduction in the free phenolic content, which is in line with a study on hawthorn [53].

Microwave drying increased the caffeic acid content to 46.45 mg/kg DW due to the ortho-dihydroxyphenolic structure (3,4-dihydroxy-substituted cinnamic acid backbone with intramolecular hydrogen bonding), thereby conferring its thermal stability [54], combined with microwave-induced cell wall disruption, thus enhancing its release [45]. This pattern aligns with observations in *Capparis spinosa* L. fruits (a 13 μg/g increase) [18], in which microwave processing selectively converted chlorogenic acid esters into free caffeic acids [17,43,44].

### 3.5. Multivariate Analysis

**Figure 3:**
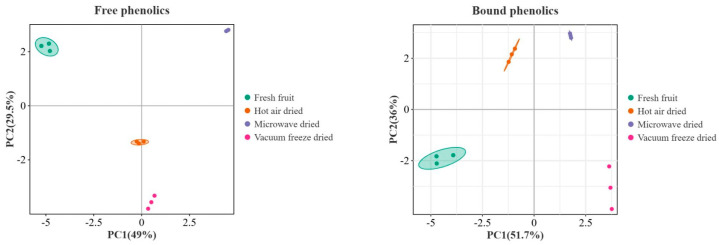
PCA plot of the phenolic profiles in Noni fruits under different drying methods.

The principal component analysis (PCA) plots in Figure 3 demonstrate the distinct distribution patterns of the free and bound phenolics in Noni fruits under different treatments. PC1 and PC2, representing composite variables, accounted for substantial variance proportions, as indicated by the percentage values. Fresh fruits (green dots) formed a unique cluster that was markedly separated from all of the processed groups in both components. Hot-air-dried (orange), vacuum freeze-dried (pink), and microwave-dried (purple) samples exhibited significant divergences from the fresh fruits in the phenolic profiles. While all of the drying methods altered the phenolic composition, microwave drying induced the most distinct modification. Hot-air and vacuum freeze-drying showed contrasting change patterns, as evidenced by the differential spatial displacements along the principal components. These findings suggest that different drying methods substantially influence the phenolic composition of Noni.

## 4. Conclusions

This study revealed significant differences in the profiles of free and bound phenolic compounds in Noni fruit under different drying methods. Free phenolics, predominantly flavonoids and hydroxycinnamic acids, showed heightened vulnerability to thermal degradation under hot-air and microwave drying, while bound phenolics—enriched in benzoic acid derivatives—exhibited hydrolysis-dependent stability. Alkaline hydrolysis outperformed acidic or enzymatic methods in liberating the bound phenolics, reflecting structural interactions within the plant matrix. Although vacuum freeze-drying preserved the phenolic diversity by mitigating thermal degradation, compositional shifts (e.g., flavonoid degradation versus acid stabilization) highlighted the nuanced impact of drying conditions on the compound-specific stability. The findings of this study provide valuable guidance for industrial processing protocols to optimize drying methods based on targeted phytochemical retention, while also serving as a scientific basis for the development of Noni-derived processed products. Future works should focus on investigating the effects of different drying methods on the bioavailability of Noni polyphenols and their interactions with gut microbiota.

## Figures and Tables

**Table 1:** Identification of the polyphenols in Noni.

No.	RT (min)	Compounds	Formula	*m*/*z* [M-H]	*m*/*z* Fragments	Identified in
1	1.67	quinic acid ^b^	C_7_H_12_O_6_	191.0561	127.0401	F, H, M, V
2	3.04	phloroglucinol ^ab^	C_6_H_6_O_3_	125.1100		F, H, M, V
3	3.05	gallic acid ^ab^	C_7_H_6_O_5_	169.0131	125.0234	H, M
4	4.87	helicid ^ab^	C_13_H_16_O_7_	329.0878	121.0284	F, H, V
5	5.07	3,4-dihydroxybenzoic acid ^ab^	C_7_H_6_O_4_	153.0181	109.0282	F, H, M, V
6	6.54	p-hydroxybenzoic acid ^ab^	C_7_H_6_O_3_	137.0231	93.0332	F, H, V
7	7.00	esculetin ^b^	C_9_H_6_O_4_	177.0183	133.0284	F, H, M
8	7.15	caffeic acid ^ab^	C_9_H_8_O_4_	179.0337	135.0438	F, H, M, V
9	7.17	vanillic acid ^ab^	C_8_H_8_O_4_	167.0340	152.0106, 123.0440	F, H, M, V
10	7.27	syringic acid ^ab^	C_9_H_10_O_5_	197.0447	182.0214	F, H, V
11	8.10	benzoic acid ^ab^	C_7_H_6_O_2_	121.0283	77.0381, 94.0281	F, H, M, V
12	8.89	rutin ^ab^	C_27_H_30_O_16_	609.1467	300.0279, 301.0354	F, H, M, V
13	8.99	p-coumaric acid ^ab^	C_9_H_8_O_3_	163.0386	119.0401	M, V
14	9.00	vanillin ^ab^	C_8_H_8_O_3_	151.0389	136.0155	F, H, M, V
15	9.02	p-hydroxycinnamic acid ^ab^	C_9_H_8_O_3_	163.0389	119.0491	F, H, M, V
16	9.32	hyperoside ^ab^	C_21_H_20_O_12_	463.0886	300.0282, 301.0355	F, H, M, V
17	9.46	isoquercitrin ^ab^	C_21_H_20_O_12_	463.0885	300.0276	F, H, M, V
18	9.74	ferulic acid ^ab^	C_10_H_10_O_4_	193.0497	178.0265, 134.0363, 149.0599	F, H, M, V
19	9.99	kaempferol-3-o-rutinoside ^b^	C_27_H_30_O_15_	593.1514	285.0404	F, H, V
20	10.29	isoferulic acid ^ab^	C_10_H_10_O_4_	193.0498	178.0265, 134.0363	F, H, M, V
21	10.60	kaempferol-3-o-glucoside ^ab^	C_21_H_20_O_11_	447.0931	284.0328	V
22	11.32	hesperidin ^b^	C_28_H_34_O_15_	609.1829	301.0723	F, H
23	13.07	salicylic acid ^b^	C_7_H_6_O_3_	137.0230		F, H, V
24	13.32	morin ^b^	C_15_H_14_O_9_	301.0359	151.0028	F, V
25	14.60	quercetin ^ab^	C_15_H_10_O_7_	301.0352	151.0030, 178.9983	F, H, M, V
26	15.65	cinnamic acid ^ab^	C_9_H_8_O_2_	147.0439	103.0536	M, V
27	16.43	kaempferol ^ab^	C_15_H_10_O_6_	285.0411	151.0014	F, H, M

^a^ compared with reference standards; ^b^ compared with references; F: fresh fruit; H: hot-air-dried; M: microwave-dried; V: vacuum freeze-dried.

**Table 2:** Contents of polyphenols in fresh fruits.

No.	Compounds	80% Methanol (mg/kg DW)	Water (mg/kg DW)	Acid (mg/kg DW)	Base (mg/kg DW)	Enzyme (mg/kg DW)
1	salicylic acid	23.15 ± 2.10	26.32 ± 2.97	3.89 ± 0.22	2.57 ± 0.57	1.20 ± 0.27
2	phloroglucinol	1.93 ± 0.70	1.99 ± 0.37	1.30 ± 0.17	13.10 ± 2.23	0.32 ± 0.09
3	3,4-dihydroxybenzoic acid	6.07 ± 0.66	7.50 ± 1.00	1.64 ± 0.34	1.36 ± 0.47	ND
4	p-hydroxybenzoic acid	44.12 ± 2.82	45.33 ± 2.42	3.47 ± 0.20	5.50 ± 0.04	2.44 ± 0.06
5	caffeic acid	0.13 ± 0.03	1.17 ± 0.22	ND	0.89 ± 0.06	ND
6	syringic acid	1.72 ± 0.20	1.47 ± 0.20	0.25 ± 0.05	0.68 ± 0.07	ND
7	benzoic acid	ND	ND	69.50 ± 7.95	220.67 ± 14.57	23.54 ± 2.02
8	rutin	1446.09 ± 21.18	1016.21 ± 14.98	0.24 ± 0.06	77.02 ± 3.25	65.97 ± 1.21
9	p-coumaric acid	ND	ND	0.74 ± 0.28	23.32 ± 0.32	ND
10	hyperoside	15.78 ± 0.36	26.49 ± 1.21	1.38 ± 0.04	3.48 ± 1.10	1.71 ± 0.32
11	isoquercitrin	12.09 ± 0.05	20.51 ± 0.24	1.09 ± 0.06	2.78 ± 0.86	1.36 ± 0.24
12	ferulic acid	3.80 ± 0.64	5.69 ± 0.24	2.46 ± 0.34	11.20 ± 0.55	0.59 ± 0.07
13	kaempferol-3-o-rutinoside	96.89 ± 3.29	66.75 ± 2.16	ND	5.72 ± 0.88	3.73 ± 0.29
14	quercetin	139.73 ± 8.51	64.43 ± 3.90	7.46 ± 0.89	2.61 ± 1.03	3.39 ± 0.70
15	kaempferol	6.81 ± 0.98	1.41 ± 0.31	ND	0.53 ± 0.03	ND
16	quinic acid	328.37 ± 10.37	336.26 ± 12.30	9.74 ± 1.02	9.68 ± 0.64	17.58 ± 0.65
17	helicid	8.91 ± 0.61	4.19 ± 0.50	ND	ND	ND
18	esculetin	3.47 ± 0.49	2.94 ± 0.47	ND	ND	ND
19	vanillic acid	28.97 ± 0.13	29.65 ± 0.44	5.28 ± 0.15	5.37 ± 0.73	3.55 ± 0.25
20	p-hydroxycinnamic acid	ND	ND	0.93 ± 0.12	21.54 ± 0.38	0.19 ± 0.03
21	isoferulic acid	24.25 ± 0.71	28.55 ± 1.24	8.33 ± 0.60	72.51 ± 2.37	4.57 ± 0.24
22	hesperidin	0.70 ± 0.08	1.39 ± 0.04	ND	ND	ND
23	vanillin	ND	ND	2.36 ± 0.19	3.62 ± 0.22	1.22 ± 0.05
24	morin total	0.54 ± 0.05 2200.33 ± 54.94	ND 1688.25 ± 45.21	ND 120.06 ± 12.68	ND 484.15 ± 30.37	ND 131.36 ± 6.49

Data are expressed by mean ± standard deviation. ND: not detected.

**Table 3:** Contents of polyphenols in hot-air-dried fruits.

No.	Compounds	80% Methanol (mg/kg DW)	Water (mg/kg DW)	Acid (mg/kg DW)	Base (mg/kg DW)	Enzyme (mg/kg DW)
1	salicylic acid	11.68 ± 0.58	11.26 ± 0.95	0.74 ± 0.13	0.65 ± 0.12	0.36 ± 0.05
2	gallic acid	ND	ND	ND	2.44 ± 0.18	ND
3	phloroglucinol	1.24 ± 0.32	3.68 ± 0.24	1.62 ± 0.29	1.55 ± 0.29	ND
4	3,4-dihydroxybenzoic acid	5.53 ± 0.90	5.00 ± 0.70	2.40 ± 0.28	0.85 ± 0.22	ND
5	p-hydroxybenzoic acid	22.03 ± 0.03	20.56 ± 1.33	0.33 ± 0.09	ND	ND
6	caffeic acid	0.15 ± 0.04	0.35 ± 0.12	ND	ND	ND
7	syringic acid	2.60 ± 0.11	2.42 ± 0.25	0.32 ± 0.02	0.52 ± 0.03	ND
8	benzoic acid	66.58 ± 3.43	68.94 ± 1.90	44.08 ± 2.95	183.52 ± 4.14	14.64 ± 0.99
9	rutin	1063.25 ± 2.38	1068.25 ± 19.60	2.00 ± 0.12	19.42 ± 0.45	7.59 ± 0.71
10	p-coumaric acid	ND	ND	ND	10.91 ± 0.41	0.18 ± 0.03
11	hyperoside	17.39 ± 0.90	22.32 ± 1.11	0.50 ± 0.01	2.20 ± 0.13	ND
12	isoquercitrin	28.34 ± 2.15	17.72 ± 1.07	0.34 ± 0.09	1.46 ± 0.39	ND
13	ferulic acid	1.75 ± 0.28	1.90 ± 0.13	2.02 ± 0.31	5.75 ± 0.73	0.91 ± 0.15
14	kaempferol-3-o-rutinoside	70.49 ± 4.31	73.20 ± 0.15	ND	0.87 ± 0.07	0.23 ± 0.03
15	quercetin	30.53 ± 4.00	3.34 ± 0.27	ND	ND	ND
16	kaempferol	ND	ND	ND	0.53 ± 0.12	ND
17	quinic acid	472.41 ± 6.21	298.64 ± 3.81	4.55 ± 0.79	6.70 ± 0.69	2.80 ± 0.59
18	helicid	1.84 ± 0.49	ND	ND	ND	ND
19	esculetin	2.65 ± 0.16	2.50 ± 0.38	ND	ND	ND
20	vanillic acid	23.30 ± 3.03	23.76 ± 1.15	4.94 ± 0.20	5.29 ± 0.31	1.62 ± 0.11
21	p-hydroxycinnamic acid	ND	ND	ND	10.31 ± 0.39	0.31 ± 0.10
22	isoferulic acid	17.44 ± 0.71	17.19 ± 1.07	6.95 ± 0.68	51.58 ± 1.00	5.33 ± 0.68
23	hesperidin	ND	0.03 ± 0.01	ND	ND	ND
24	vanillin	2.98 ± 0.02	2.86 ± 0.19	1.98 ± 0.07	3.08 ± 0.06	ND
	total	1842.18 ± 30.05	1620.16 ± 34.43	72.77 ± 6.03	307.63 ± 9.73	33.97 ± 3.44

Data are expressed by mean ± standard deviation. ND: not detected.

**Table 4:** Contents of polyphenols in the microwave-dried fruits.

No.	Compounds	80% Methanol (mg/kg DW)	Water (mg/kg DW)	Acid (mg/kg DW)	Base (mg/kg DW)	Enzyme (mg/kg DW)
1	gallic acid	1.22 ± 0.16	0.26 ± 0.08	2.66 ± 0.03	8.44 ± 0.28	0.85 ± 0.03
2	phloroglucinol	3.35 ± 0.48	0.71 ± 0.07	3.38 ± 0.63	7.49 ± 0.29	0.50 ± 0.18
3	3,4-dihydroxybenzoic acid	1.19 ± 0.08	1.14 ± 0.29	1.00 ± 0.22	1.57 ± 0.13	ND
4	p-hydroxybenzoic acid	4.91 ± 0.93	4.29 ± 0.33	12.97 ± 1.17	14.27 ± 1.47	4.46 ± 0.24
5	caffeic acid	46.45 ± 1.20	32.21 ± 2.62	ND	ND	ND
6	benzoic acid	13.30 ± 0.90	14.54 ± 2.11	21.65 ± 2.00	118.21 ± 0.40	11.25 ± 1.82
7	rutin	609.72 ± 7.21	465.18 ± 11.40	0.24 ± 0.04	5.84 ± 0.08	4.40 ± 0.16
8	p-coumaric acid	ND	ND	ND	10.01 ± 0.13	ND
9	hyperoside	16.35 ± 0.70	11.00 ± 0.51	0.35 ± 0.05	0.59 ± 0.09	0.23 ± 0.02
10	isoquercitrin	22.69 ± 1.02	15.84 ± 1.81	0.53 ± 0.03	0.27 ± 0.12	0.37 ± 0.00
11	ferulic acid	0.80 ± 0.06	0.61 ± 0.06	0.72 ± 0.05	4.02 ± 0.14	0.22 ± 0.04
12	quercetin	25.90 ± 0.38	1.66 ± 0.09	0.77 ± 0.01	ND	ND
13	cinnamic acid	ND	ND	3.41 ± 0.17	ND	ND
14	kaempferol	0.47 ± 0.06	ND	1.03 ± 0.05	ND	ND
15	quinic acid	68.35 ± 0.42	91.20 ± 0.56	1.69 ± 0.10	2.08 ± 0.07	9.85 ± 0.50
16	esculetin	1.44 ± 0.06	1.08 ± 0.03	0.26 ± 0.02	ND	0.03 ± 0.01
17	vanillic acid	3.53 ± 0.01	2.78 ± 0.08	2.13 ± 0.16	ND	1.04 ± 0.01
18	p-hydroxycinnamic acid	ND	ND	ND	9.78 ± 0.25	ND
19	isoferulic acid	3.41 ± 0.52	2.93 ± 0.05	2.36 ± 0.04	23.22 ± 0.20	1.48 ± 0.18
20	vanillin	2.36 ± 0.00	ND	1.67 ± 0.10	3.01 ± 0.01	1.16 ± 0.00
	total	825.44 ± 14.19	645.43 ± 20.09	55.82 ± 4.87	208.80 ± 3.66	35.84 ± 3.19

Data are expressed by mean ± standard deviation. ND: not detected.

**Table 5:** Contents of polyphenols in the vacuum freeze-dried fruits.

No.	Compound	80% Methanol (mg/kg DW)	Water (mg/kg DW)	Acid (mg/kg DW)	Base (mg/kg DW)	Enzyme (mg/kg DW)
1	salicylic acid	12.67 ± 0.69	12.25 ± 0.79	1.83 ± 0.29	1.50 ± 0.17	0.44 ± 0.04
2	phloroglucinol	2.63 ± 0.10	1.39 ± 0.08	1.68 ± 0.32	9.53 ± 0.47	ND
3	3,4-dihydroxybenzoic acid	0.40 ± 0.02	1.33 ± 0.04	1.85 ± 0.11	0.47 ± 0.06	ND
4	p-hydroxybenzoic acid	10.39 ± 0.34	15.50 ± 0.61	46.89 ± 1.07	1.43 ± 0.04	ND
5	caffeic acid	ND	ND	ND	2.67 ± 0.17	ND
6	syringic acid	1.77 ± 0.07	1.52 ± 0.10	0.40 ± 0.07	0.34 ± 0.08	ND
7	benzoic acid	35.56 ± 0.53	34.13 ± 0.68	50.71 ± 1.08	209.97 ± 2.34	28.48 ± 0.45
8	rutin	1809.83 ± 14.92	1076.00 ± 26.96	1.85 ± 0.02	34.31 ± 1.84	5.78 ± 0.50
9	p-coumaric acid	ND	ND	0.78 ± 0.02	20.38 ± 1.01	ND
10	hyperoside	124.55 ± 5.78	59.62 ± 2.05	1.16 ± 0.20	3.07 ± 0.76	ND
11	isoquercitrin	101.32 ± 2.27	46.79 ± 1.17	0.94 ± 0.10	2.04 ± 0.34	0.08 ± 0.01
12	ferulic acid	1.23 ± 0.13	1.64 ± 0.18	1.41 ± 0.15	8.92 ± 0.94	0.41 ± 0.05
13	kaempferol-3-o-rutinoside	153.53 ± 1.71	64.58 ± 1.08	ND	1.84 ± 0.11	0.32 ± 0.05
14	kaempferol-3-o-glucoside	2.84 ± 0.10	1.29 ± 0.07	ND	ND	ND
15	quercetin	13.55 ± 0.43	3.39 ± 0.33	1.08 ± 0.06	ND	ND
16	cinnamic acid	ND	ND	ND	ND	4.73 ± 0.34
17	quinic acid	198.72 ± 2.98	286.55 ± 1.51	12.90 ± 0.78	8.69 ± 0.30	7.75 ± 0.65
18	helicid	6.88 ± 0.14	4.79 ± 0.35	ND	ND	ND
19	vanillic acid	14.19 ± 0.60	13.56 ± 0.21	5.56 ± 0.17	2.36 ± 0.08	1.91 ± 0.15
20	p-hydroxycinnamic acid	ND	ND	0.82 ± 0.18	19.01 ± 0.86	0.15 ± 0.02
21	isoferulic acid	13.43 ± 0.39	11.58 ± 0.64	4.89 ± 0.72	51.03 ± 0.82	5.13 ± 0.37
22	vanillin	2.59 ± 0.07	2.62 ± 0.07	3.79 ± 0.41	3.77 ± 0.09	1.21 ± 0.05
23	morin total	0.48 ± 0.02 2506.56 ± 31.29	ND 1638.53 ± 36.92	ND 138.54 ± 5.75	ND 390.25 ± 11.48	ND 56.39 ± 2.68

Data are expressed by mean ± standard deviation. ND: not detected.

## Data Availability

The original contributions presented in the study are included in the article/Supplementary Material; further inquiries can be directed to the corresponding author.

## Supplementary Materials

The following supporting information can be downloaded at: https://www.mdpi.com/article/10.3390/foods14081398/s1.
