# Supplementary material for: The Comparison of the Profile of Phenolic Compounds in Noni (Morinda citrifolia L.) Fruit by Different Drying Methods
Source: Foods. 2025 Apr 17;14(8):1398. doi: 10.3390/foods14081398 (PMC12026537; doi:10.3390/foods14081398)
Supplement: Supplementary file 1 [file foods-14-01398-s001.zip › foods-3558425-supplementary.pdf]

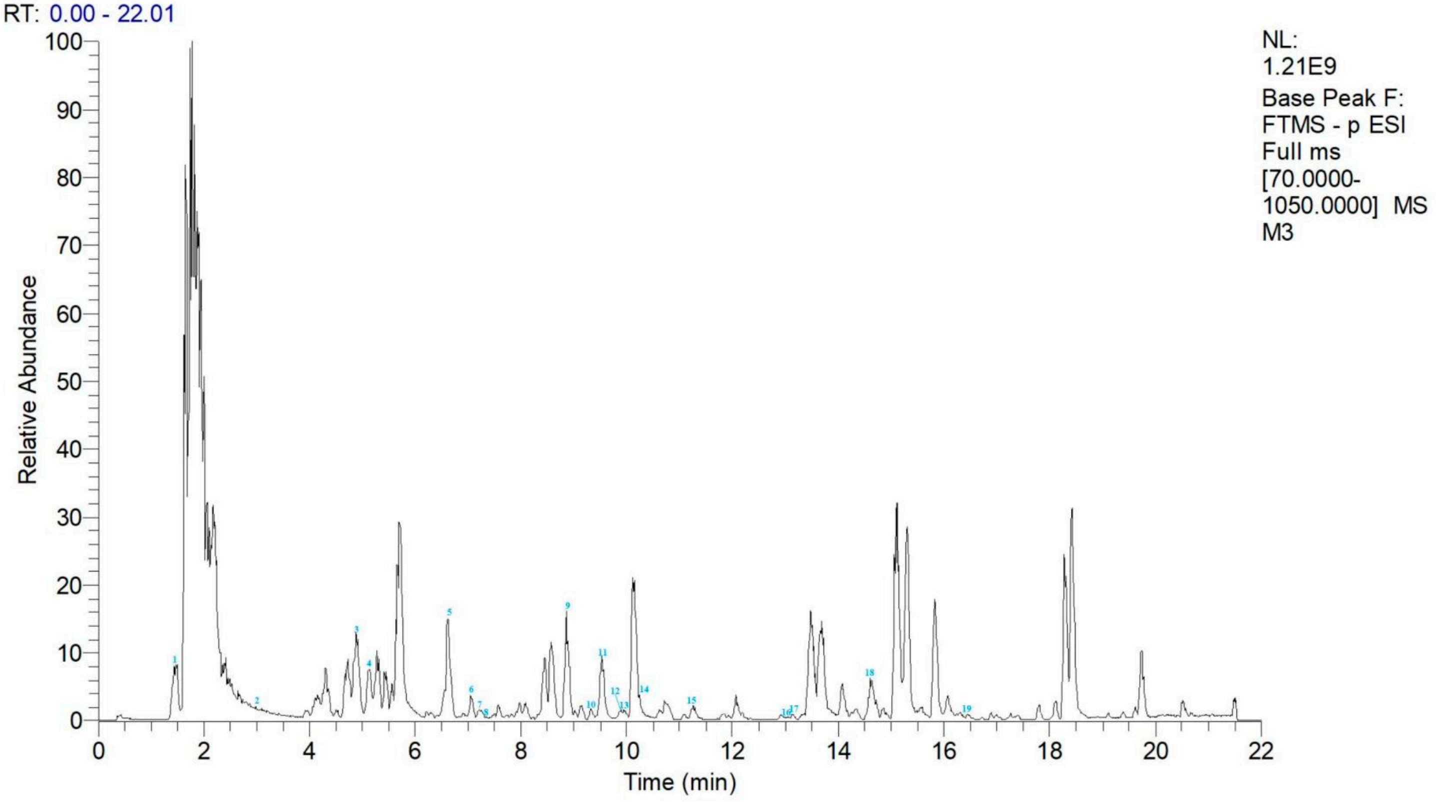

Figure S1 The ion chromatograms of fresh fruits.

1: quinic acid; 2: phloroglucinol; 3: helicid; 4: 3,4-dihydroxybenzoic acid; 5: p-hydroxybenzoic acid; 6: esculetin; 7: vanillic acid; 8: syringic acid; 9: rutin; 10: hyperoside; 11: isoquercitrin; 12: ferulic acid; 13: kaempferol-3-O-rutinoside; 14: isoferulic acid; 15: hesperidin; 16: salicylic acid; 17: morin; 18: quercetin; 19: kaempferol.

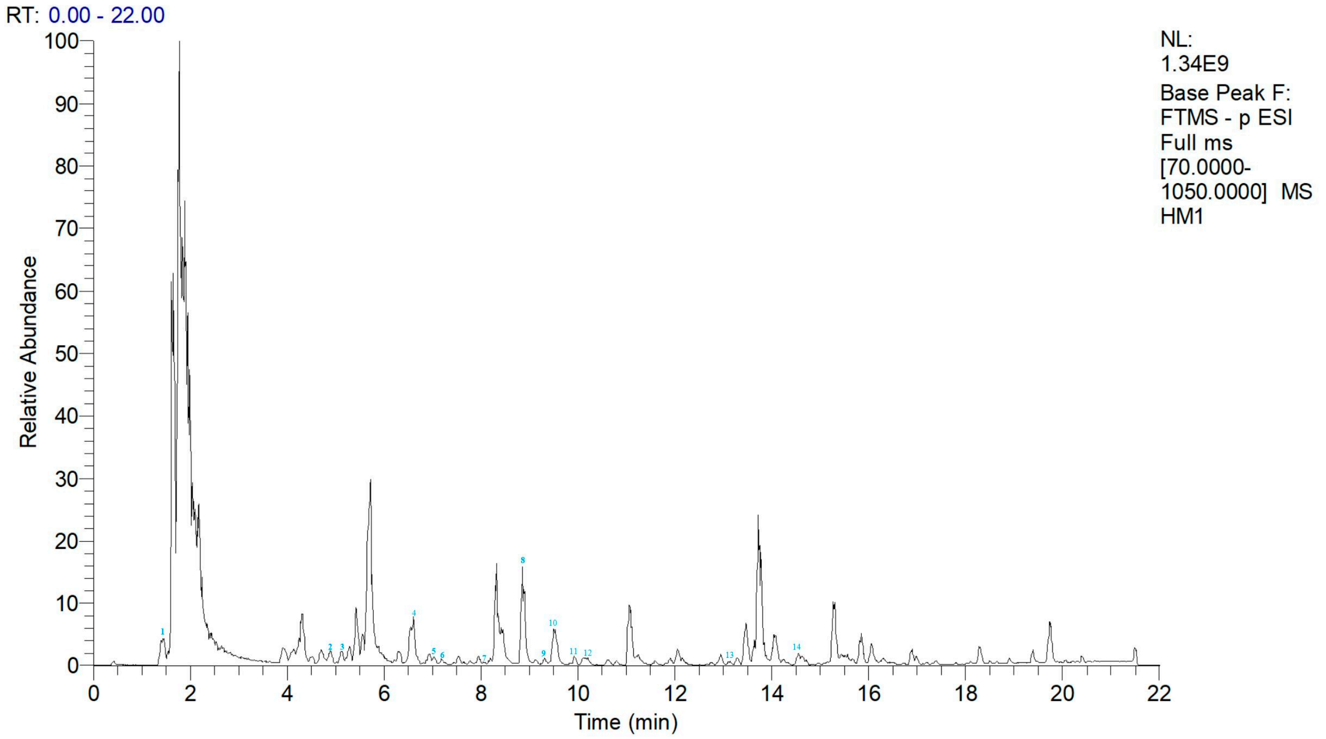

Figure S2 The ion chromatograms of hot air dried fruits.

1: quinic acid; 2: helicid; 3: 3,4-dihydroxybenzoic acid; 4: p-hydroxybenzoic acid; 5: esculetin; 6: vanillic acid; 7: benzoic acid; 8: rutin; 9: hyperoside; 10: isoquercitrin; 11: kaempferol-3-O-rutinoside; 12: isoferulic acid; 13: salicylic acid; 14: quercetin.

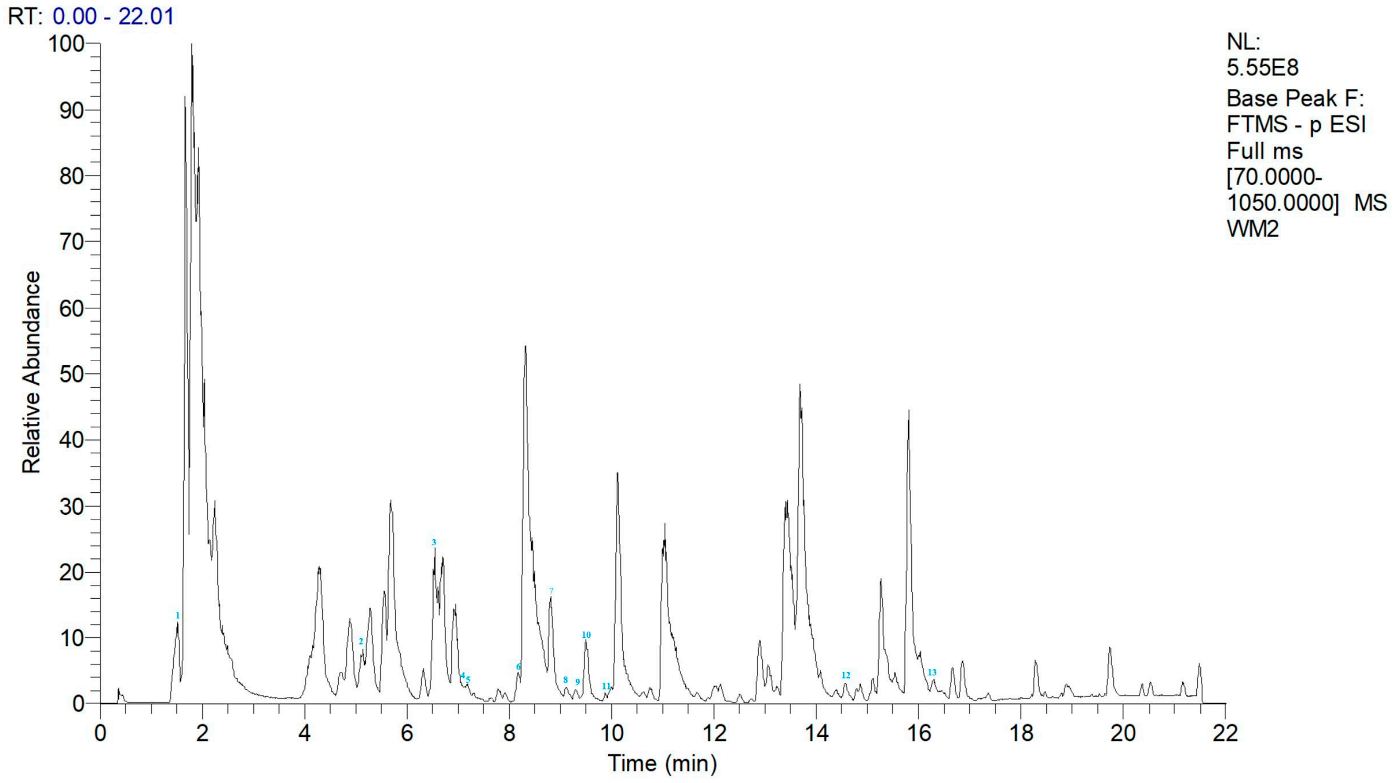

Figure S3 The ion chromatograms of microwave dried fruits.

1: quinic acid; 2: 3,4-dihydroxybenzoic acid; 3: p-hydroxybenzoic acid; 4: esculetin; 5: caffeic acid; 6: benzoic acid; 7: rutin; 8: vanillin; 9: hyperoside; 10: isoquercitrin; 11: ferulic acid; 12: quercetin; 13: kaempferol.

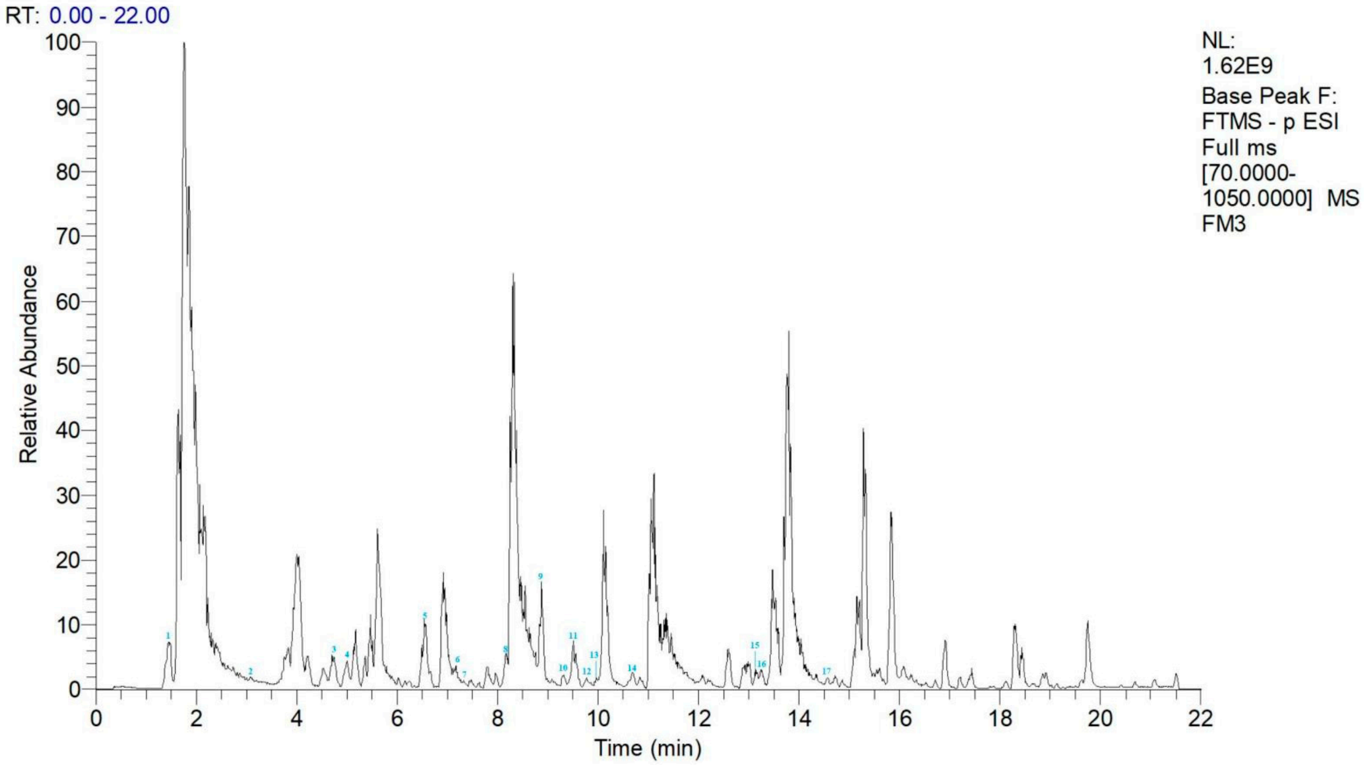

Figure S4 The ion chromatograms of vacuum freeze dried fruits.

1: quinic acid; 2: phloroglucinol; 3: helicid; 4: 3,4-dihydroxybenzoic acid; 5: p-hydroxybenzoic acid; 6: vanillic acid; 7: syringic acid; 8: benzoic acid; 9: rutin; 10: hyperoside; 11: isoquercitrin; 12: ferulic acid; 13: kaempferol-3-O-rutinoside; 14: kaempferol-3-O-glucoside;15: salicylic acid; 16: morin; 17: quercetin.
